# Patients with total hip arthroplasty were more physically active 9.6 years after surgery: a case-control study of 429 hip arthroplasty cases and 29,272 participants from a population-based health study

**DOI:** 10.2340/17453674.2024.40815

**Published:** 2024-05-30

**Authors:** Jakob Vangen NORDBØ, Truls M STRAUME-NÆSHEIM, Geir HALLAN, Anne Marie FENSTAD, Einar Andreas SIVERTSEN, Asbjørn ÅRØEN

**Affiliations:** 1 Department of Orthopedic Surgery, Akershus University Hospital, Lørenskog; Institute of Clinical Medicine, Campus Ahus, University of Oslo, Oslo; 2 Department of Orthopedic Surgery, Haukeland University Hospital, Bergen; Department of Clinical Medicine (K1), University of Bergen, Bergen; 3 The Norwegian Arthroplasty Register, Department of Orthopedic Surgery, Haukeland University Hospital, Bergen; 4 Department of Orthopedic Surgery, Lovisenberg Diaconal Hospital, Norway

## Abstract

**Background and purpose:**

Few studies report on long-term levels of physical activity after THA compared with a control population. This case-control study aimed to find the long-term habitual level of leisure-time physical activity after THA and compare it with a large control group.

**Patients and methods:**

A randomized sample of 856 patients, treated with primary THA, were identified from the Norwegian Arthroplasty Register. 429 (50%) responded to a questionnaire with a mean follow-up time of 9.6 years. We compared them with a control group of 29,272 (64%) from a population-based health study. Physical activity was measured with a questionnaire and categorized into groups according to the general recommendations for physical activity.

**Results:**

245 (63%) of the THA cases reported a level of leisure-time physical activity meeting the general recommendations, compared with 10,803 (39%) in the control group. The difference persisted at all ages (50–90 years). In sex, age, and BMI-adjusted regression models the chance of meeting the physical activity recommendations was higher in the THA group than in the control group (OR 2.9, 95% confidence interval 2.4–3.6).

**Conclusion:**

The majority of the patients with THA reported a level of leisure-time physical activity meeting the general recommendations for physical activity. THA patients were more physically active in their leisure time than a control group representing a normal population.

Regular physical activity is associated with improved quality of life and a wide range of health benefits [1]. It is recommended for adults of all ages to do at least 150–300 minutes a week of moderate-intensity or 75–150 minutes a week of vigorous-intensity aerobic physical activity to reduce all-cause mortality and morbidity [2]. Patients with osteoarthritis (OA) experience pain as the most disabling symptom leading to reduced physical activity levels [3]. According to a WHO citation from 2015, globally, 80% of individuals with OA will have limitations in movement [4].

While it is known that total hip arthroplasty (THA) relieves pain and increases physical function in patients with end-stage OA, there is only a small to moderate improvement in observed physical activity in the first 6–12 months after THA [5]. After THA, patients are more likely to return to low-intensity activities such as walking and cycling than high-intensity activities such as running and tennis [6]. Physical activity levels in the years following THA seem to be low, with less than half of subjects meeting health-enhancing guidelines [7]. Few studies report on long-term levels of physical activity after THA compared with a control population [8]. Considering the benefits of physical activity and the increasing numbers of THA in younger and more physically active patients (< 65 years old) [9], it is important to know the long-term physical activity level in THA patients.

The purpose of this study was to explore the achievable long-term level of physical activity after THA and to compare it with a control group representing a normal population.

## Methods

### Study design and data sources

This is a case-control study based on data from the Norwegian Arthroplasty Register (NAR), patient-reported outcomes (PROMS) and the Trøndelag Health Study (HUNT) reported according to the STROBE guidelines [10]. Norwegian surgeons register data on primary THAs in NAR with a completeness of 97% [11]. The collected data includes information on the patient’s age, sex, date of the operation, American Society of Anesthesiologists classification (ASA class), indication for surgery, type of surgical procedure, surgical approach, fixation method, and time spent on surgery. The HUNT Study is a large population-based health study conducted in a region in the middle of Norway. It collects questionnaire data, clinical measurements and biological samples. All residents above 18 years of age are invited. In this study, we use data from the third wave of HUNT [12].

### Study population

Patients eligible for the study had unilateral primary THAs with articulations made of a metal or ceramic femoral head on a highly cross-linked polyethylene counter surface (HXLPE). HXLPE was introduced in Norway in 2005. To study patients with reasonably high physical activity at a long-term follow up we selected patients aged 40–75 years at the time of surgery, registered in 2005–2012 [13]. Patients revised before the end of the study were excluded. Identified patients were asked to fill out a questionnaire sent by mail in December 2020. Non-responders received a reminder by mail in February 2021. A second reminder was sent as a text message by phone in July 2021.

To match the THA population at the age of data collection we selected a control population of participants from the HUNT3 Survey aged 50–90 years. The HUNT3 Survey was carried out in 2006–2008. The HUNT3 cohort is previously described in more detail elsewhere [12].

### Outcome

**Figure 1:**
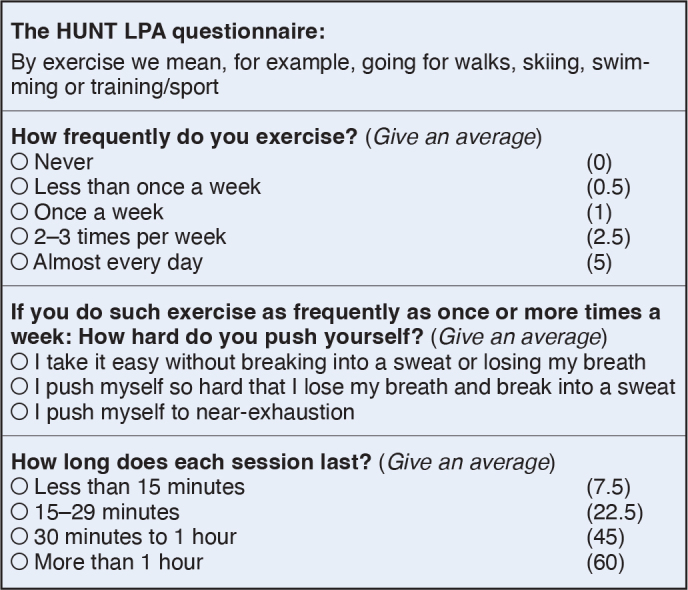
The HUNT LPA questionnaire. Numbers in parentheses indicate the score used for each response when calculating the LPA categories. LPA = leisure-time physical activity.

Participants reported their leisure-time physical activity levels by answering the previously validated HUNT LPA questionnaire [14], which contains questions on the frequency, intensity, and duration of exercise (Figure 1). The answer option “I take it easy without breaking into a sweat or losing my breath” was considered as moderate intensity and the options “I push myself so hard that I lose my breath and break into a sweat” or “I push myself to near-exhaustion” were considered as high intensity. We multiplied weighted scores of frequencies and duration of physical activity and categorized it into moderate or high-intensity physical activity. From this we made the 4 categories inactive, low, moderate, and high leisure-time physical activity. To perform the statistical analysis, a dichotomous variable, meeting or not meeting the general recommendations for physical activity, was constructed according to the recommendations from the European Society of Cardiology (ESC) [2,15] (Table 1).

**Table 1:** Categories of leisure-time physical activity (LPA) according to the general PA recommendations

Item	Minutes of LPA per week	
	Moderate intensity	Vigorous intensity
Not meeting the recommendations		
Inactive	None	None
Low	< 150	< 75
Meeting the recommendations		
Moderate	150–299	75–149
High	≥ 300	≥ 150

### Statistics

Descriptive data is presented as means with standard deviations (SD), compared using the independent samples t-test, for continuous variables and numbers with percentages (%), compared using the chi-squared test, for categorical variables. We investigated the coherence of leisure-time physical activity between the study groups at increasing age, with 95% confidence intervals (CI). We performed logistic regression analyses to study the association between the 2 study groups and the chance of meeting the leisure-time physical activity level adjusted for age, sex, and BMI. All statistical analyses were performed using Stata 17.0 SE standard edition (StataCorp LLP, College Station, TX, USA). All tests were 2-sided and a P value < 0.05 was considered statistically significant.

### Sensitivity analyses

To verify the robustness of the measured categories of leisure-time physical activity we performed a linear regression between the study groups on the previously validated LPA index, which is a continuous variable formed by the product of frequency, duration, and intensity of leisure-time physical activity [14]. We performed multiple imputation analyses on the dependent variable of leisure-time physical activity to include a value for missing observations among the responders in the regression analyses. We also conducted single imputation analyses on the missing leisure-time physical activity data of the non-responders, predicting a worst- and best-case scenario. In the worst-case scenario, missing data was replaced by inactive levels and in best-case scenario by high levels of leisure-time physical activity.

### Ethics, funding, and disclosures

The regional committee for medical and health research ethics approved the study (2019/44572/REK sør-øst A). All participants in the NAR and the HUNT study gave informed written consent before participating. Based on this consent the NAR has permission from the Norwegian Data Inspectorate to collect patient data (ref 24.1.2017: 16/01622-3/CDG). The project received a research grant from Ortomedic AS and Eckbos legate. The authors declare no conflicts of interest for this work. Complete disclosure of interest forms according to ICMJE are available on the article page, doi: 10.2340/17453674.2024.40815

## Results

**Figure 2:**
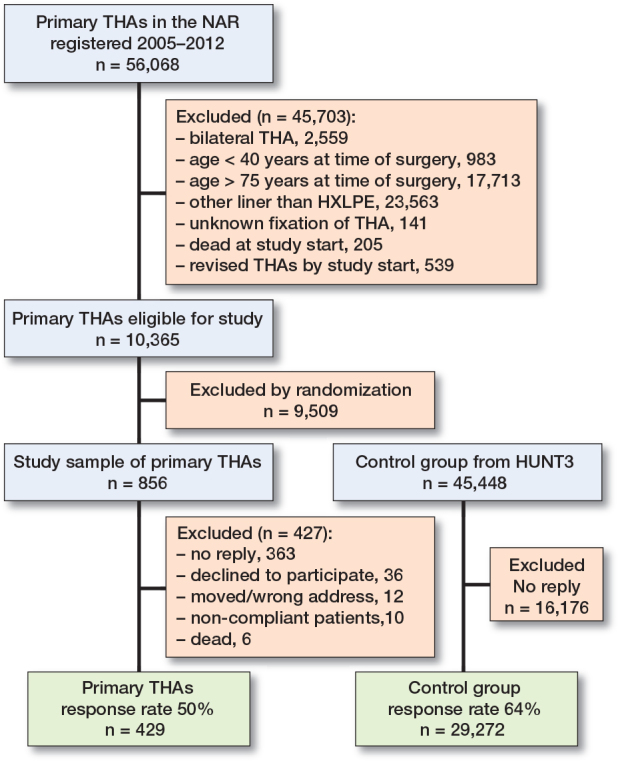
Flowchart of the selection of eligible patients from the Norwegian Arthroplasty Register (NAR), the inclusion process of the sample of patients with primary total hip arthroplasty (THA), and the inclusion process of the control group from the Trøndelag Health Study, a population health study in Norway (HUNT). HXLPE = highly cross-linked polyethylene.

429 (50%) of the sampled THA cases from the NAR and 29,701 (64%) of the HUNT participants joined the study. The selection process is illustrated in the flowchart in Figure 2.

### THA responders vs. non-responders

On average, the patients who responded in the THA group were 1.5 years younger, healthier (lower ASA class), and the proportion of males was slightly higher than among the non-responders. Osteoarthritis was the dominating indication for surgery and the implants were mostly uncemented or reversed hybrid through the lateral or the posterior approach in both responders and non-responders (Table 2, see Appendix)

**Table 2:** Comparison of descriptive characteristics of responders and non-responders in the THA group, data from the NAR. Values are count (%) unless otherwise specified

Characteristic	Responders n = 429	Non- responders n = 427	Difference between groups (CI)
Mean age (SD) ^a^	62.0 (7.4)	63.5 (8.5)	–1.5 (–2.6 to –0.4) ^b^
Female sex	247 (58)	282 (66)	–8% (–15 to –2) ^c^
ASA score			
1	154 (36)	89 (21)	15% (11 to 26) ^c^
2	240 (56)	277 (65)	–9% (–16 to –3) ^c^
≥ 3	29 (6.8)	56 (13)	–6% (–10 to –2) ^c^
Not reported	6 (1.4)	5 (1.2)	0% (–3 to 3) ^c^
Indication for surgery			
Osteoarthritis	348 (81)	331 (78)	3% (–2 to 9) ^c^
Hip dysplasia	44 (10)	39 (9.1)	1% (–3 to 5) ^c^
Acute femoral neck fractures	7 (1.6)	14 (3.3)	–1% (–4 to 1) ^c^
Other ^d^	30 (7.0)	43 (10)	–3% (–7 to 1) ^c^
Surgical approach			
Anterior (Smith–Petersen)	42 (10)	37 (8.7)	1% (–3 to 5) ^c^
Anterolateral	59 (14)	40 (9.4)	5% (1 to 9) ^c^
Lateral	195 (45)	201 (47)	–2% (–8 to 5) ^c^
Posterior	121 (28)	144 (34)	–6% (–12 to 1) ^c^
Not reported	12 (2.8)	5 (1.2)	2% (–1 to 3) ^c^
Fixation method			
Cemented	66 (15)	92 (21)	–6% (–11 to –1) ^c^
Uncemented	152 (35)	145 (34)	1% (–5 to 8) ^c^
Hybrid	13 (3.0)	3 (0.7)	2% (1 to 4) ^c^
Reversed hybrid	198 (46)	187 (44)	2% (–4 to 9) ^c^
Duration of surgery in minutes (SD)	81.8 (24.5)	84.8 (27.7)	–3.2 (–6.6 to 0.5) ^b^

a Age at time of surgery.

b Independent-samples t-test.

c Chi-squared test.

d Other indications includes, rheumatoid arthritis, Bechterew’s disease, Calve–Legg–Perthes disease, proximal epiphysiolysis of the femur, and other indications.

ASA = American Society of Anesthesiologists; also see Table 3 for abbreviations.

### THAs vs. control group

Patients answered the questionnaire from 7 to 15 years after the date of surgery, with a mean follow-up time of 9.6 (SD 1.6) years. On average, the THA patients were 7.4 years older and had a slightly lower BMI than the control group. The sex distribution between the groups was equal (Table 3).

**Table 3:** Comparison of the descriptive characteristics by study groups

Characteristic	THA group n = 429	Control group n = 29,272	Difference between groups (CI)
Mean age (SD) ^a^	71.6 (7)	64.3 (10)	7.3 (6.5 to 8.3) ^b^
Female sex, n (%)	247 (58)	15,556 (53)	5% (–1 to 9) ^c^
BMI (SD)	26.3 (4)	27.6 (4)	–1.3 (–1.7 to –0.8) ^b^

a Age at data collection.

b Independent samples t-test.

c Chi-squared test.

THA = total hip arthroplasty.

### Leisure-time physical activity

**Figure 3:**
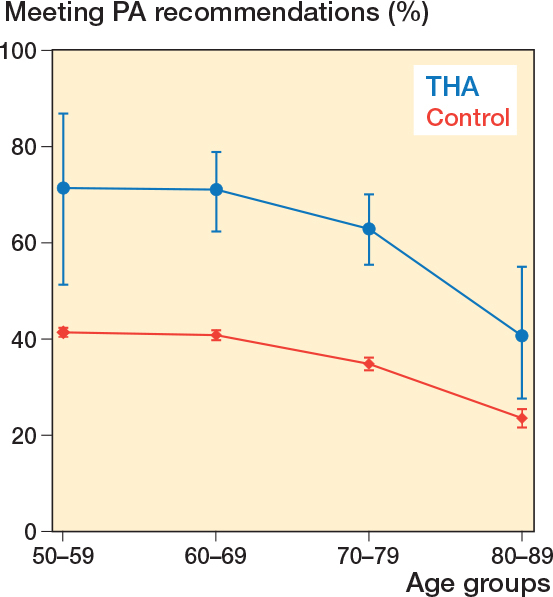
The line chart shows the coherence of meeting the general physical activity (PA) recommendations at increasing ages between the total hip arthroplasty (THA) group and the control group. The leisure-time physical activity levels are shown as the proportion (%) meeting the general PA recommendation with 95% confidence intervals.

245 (63%) of the THA patients reported a level of leisure-time physical activity meeting the general recommendations compared with 10,803 (39%) in the control group (Table 4). The level of physical activity decreased with increasing age and the difference between groups was significant in all age groups (Figure 3). Divided into the 4 categories inactive, low, moderate, and high leisure-time physical activity, there were more inactive and low-activity participants in the control group and more moderate and high-activity participants in the THA group (Table 4). In logistic regression analyses adjusted for sex, age, and BMI the chance of meeting the recommendations for physical activity was higher in the THA group than in the control group (OR 2.9, CI 2.4–3.6). Male sex, younger age, and lower BMI were associated with higher levels of physical activity.

**Table 4:** Level of leisure-time physical activity (LPA) by study groups after exclusion of incomplete questionnaires. Values are count (%) unless otherwise specified

LPA	THA group n = 388	Control group n = 29,272	Difference between groups (CI)
Index ^a^	4.0 (3.1)	2.4 (2.5)	1.6 (1.3 to 1.8) ^b^
In 4 categories			
Inactive	12 (3.1)	6,086 (22)	–19% (–21 to –17) ^c^
Low	131 (34)	11,043 (40)	–6% (–11 to –1) ^c^
Moderate	110 (28)	6,258 (22)	6% (1 to 10) ^c^
High	135 (35)	4,545 (16)	19% (14 to 23) ^c^
Dichotomized (meeting recommendations) ^d^			
	245 (63)	10,803 (39)	24% (20 to 29) ^c^

a LPA index, mean (SD): Validated LPA index, which is a continuous variable formed by the product of frequency, duration, and intensity of leisure-time physical activity (LPA) (14).

b Independent-samples t-test.

c Chi-squared test.

d Dichotomized LPA level: meeting or not meeting the general PA recommendations.

### Sensitivity analyses

We analyzed the level of leisure-time physical activity as a continuous index [13], being 4.0 (SD 3.1) in the THA group and 2.4 (SD 2.5) in the control group (Table 4). In linear regression analyses adjusted for sex, age, and BMI, being in the THA group was associated with higher LPA index compared with the control group (β 1.7, CI 1.4–1.9).

The HUNT LPA questionnaire was incomplete for 41 participants (10%) in the THA group and 1,340 (5%) in the control group. We performed multiple imputation on the missing values of leisure-time physical activity with 10 imputations of each missing value. In logistic regression analyses of the imputed leisure-time physical activity level, adjusted for age and sex, the chance of meeting the recommendations for physical activity was still higher in the THA group than in the control group (OR 3.1, CI 2.5–3.9). In the single imputation analyses on the missing values of leisure-time physical activity from non-responders, both the worst- and best-case scenario supported the conclusion that the chance of meeting the recommendations for physical activity was higher in the THA group (Table 5, see Appendix).

**Table 5:** Sensitivity analysis: level of leisure-time physical activity (LPA) by study groups after multiple and single imputation of missing values on LPA in the groups

	LPA dichotomized: Meeting PA recommendations				OR (CI) ^a^	Difference between groups (CI) ^b^
	THA group		Control group			
	total n	n (%)	total n	n (%)		
Observed data	388	245 (63)	27,932	10,803 (39)		24% (20–29)
Multiple imputation ^c^	429	N/A	29,272	N/A	2.7 (2.1–3.3)	N/A
Single imputation,						
Worst case ^d^	856	245 (29)	45,448	10,803 (24)		5% (2–8)
Best case ^e^	856	713 (83)	45,448	28,319 (62)		21% (18–24)

a Logistic regression analyses: crude model with OR of the multiple imputed LPA level between the THA group and the control group.

b Chi-squared test.

c 10 imputations of each missing LPA value among responders with incomplete questionnaires, 41 participants in the THA group and 1,340 in the control group.

d Single imputation where missing data is replaced by inactive LPA levels among all participants eligible for the study in both groups.

e Single imputation where missing data is replaced by high LPA levels among all participants eligible for the study in both groups.

N/A, not applicable; also see Table 3 for abbreviations.

## Discussion

The aim of this study was to explore the long-term level of leisure-time physical activity after THA. Patients with THA showed a higher level of leisure-time physical activity 7 to 15 years after surgery, and were more likely to meet the health-enhancing recommendations for physical activity, compared with a representative control population.

Our study reports higher levels of leisure-time physical activity in the THA group than previously described [7]. This may be explained by selection. We selected a “middle-aged” population with THA, with a mean age of 63 years at the time of surgery, to be able to study long-term levels of leisure-time physical activity. The mean age for having a THA in Norway was 70 years for women and 68 years for men in 2022, making our sample a bit younger than the unselected THA population [11]. Compared with the non-responders the responding THAs had less comorbidity according to ASA class, slightly younger age, and were less likely to be females. Younger age and less comorbidity are predictors of increased physical functioning after THA [13]. This might have introduced selection bias considering the high proportion meeting the general recommendations for physical activity in the THA group and thus may have influenced the external validity of our results.

In a cross-sectional study of 273 primary THA cases compared with a normal population, Wagenmakers et al. found no significant difference in the total amount of physical activity at a mean of 3.3 years after the operation. The national guidelines were met by 51% of the participants with THA and 49% of the controls [16]. Our control group reported lower levels of physical activity than the control group from the study of Wagenmakers et al., which may be explained by the slightly higher age in our study with a mean of 64.3 years compared with 62.4 years. Unfortunately, we do not possess a comparable comorbidity scale between the studies, but the control group of Wagenmakers et al. consisted of age- and sex-matched “healthy” counterparts whereas our control group was matched only on age and unselected on comorbidities. In our study, the control group had a slightly higher BMI than the THA group, which is a known negative predictor of physical functioning [13]. Despite being 7.4 years younger than the THA group at the time of data collection, the control group was less physically active than the THA group. However, our control group was part of the HUNT3 cohort, which did not possess more comorbidity than the average in the eligible population. Published results of nonparticipants in HUNT3 show that they had even lower socioeconomic status, higher mortality, and higher prevalence of cardiovascular diseases, diabetes mellitus, and psychiatric disorders than participants did [17].

Patients at increased risk of early mortality are less likely to undergo surgery [18]. This selection can be transferable to the reduced mortality seen in the first decade after THA [19]. Combined with the exclusion of THA patients registered with revision surgery, this might have introduced a “healthy worker effect”, selecting a healthier population, in our THA group. However, this study indicates that THA enables this population to maintain their level of physical activity. Nevertheless, selection bias and the low response rate of the study could affect the robustness of our results. In the sensitivity analyses, we showed a “worst-case scenario,” with imputed inactive levels of physical activity on all missing data on leisure-time physical activity. The THA group was still more likely to meet the general recommendation of physical activity than the control group. Thus, we conclude that the result is reliable despite the missing values for leisure-time physical activity.

Though the HUNT LPA questionnaire is utilized for epidemiological research [14], self-reported physical activity is known to be overestimated due to recall bias [20]. In a cross-sectional study of 4,867 Americans, Troiano et al. found that less than 5% of adults met the criteria of recommendations based on accelerometer-derived results compared with 51% in self-reported questionnaires from the same national survey [21]. Accelerometers only give a snapshot of physical activity and are likely to underestimate certain activities such as cycling, swimming, or upper body movement [20]. The HUNT LPA questionnaire was not designed to estimate physical activity according to the recommendations for physical activity. The questionnaire does not include housework or occupational activity, which may have underestimated the level of physical activity in participants doing manual labor. We still chose this method because of the known importance of physical activity in improving the quality of life [1].

### Strengths

One strength of our study is the large control group. The population is in most respects representative of Norway with the exception that the geographic region has no larger cities and therefore the population is slightly more rural than the average national population [22]. The THA cases, on the other hand, make up a random national sample.

### Limitations

The low response rate of the THA cases is a limitation, though it was in the range of expected response rates of long-term follow-up registry studies [23]. Fortunately, the NAR possesses baseline data on the non-responders, which deduces the representativeness of our sample. In a study from the Australian Orthopedic Association National Joint Replacement Registry, Harris et al. found small differences between responders and non-responders on baseline data when they were asked to fill out PROMs electronically. An additional phone call to non-responders showed no significant difference for most outcomes compared with electronic responders, and the author concluded that electronic-only follow-up on PROMs after THA seems to provide a satisfactory representation of the population invited to participate [24].

The lack of a comparable comorbidity scale between the THA and the control group is a limitation. In addition, the THA cases were recruited from the whole country, whereas the control group was from a specific region. Though a case-control study design is fit to answer the research question of this study, a longitudinal design with access to preoperative levels of physical activity in the THA group could have described change on an individual level. Fortunately, the NAR started electronic follow-up on PROMs preoperatively in 2017, with intended follow-up at 1, 6, and 10 years post-surgery. This enables us to study the pre- and post-levels of physical activity in THA patients in more detail in the near future.

### Conclusion

We found that OA patients with THA were more physically active in their leisure time, at a mean of 9.6 years after surgery, than a control group representing a normal population. Based on these findings, OA patients awaiting THA at an age of 40–75 years have a chance to achieve a long-term level of physical activity meeting the health-enhancing recommendations for physical activity. This is important knowledge for surgeons advising OA patients into having THA and for health authorities promoting general health in an aging population.

## Notes

All authors participated in the conceptualization of the study. JN collected the patient reported outcome measures from the THA cases, performed the data analysis, and wrote the original draft of the manuscript. All authors interpreted the results, reviewed and edited the manuscript.

The authors thank the orthopedic surgeons of Norway for their excellent reporting of joint replacement surgeries to the national register. The Trøndelag Health Study (HUNT) is a collaboration between HUNT Research Centre (Faculty of Medicine and Health Sciences, Norwegian University of Science and Technology NTNU), Trøndelag County Council, Central Norway Regional Health Authority, and the Norwegian Institute of Public Health. Finally, they thank Hanna Vangen for her critical appraisal and help with preparing the data. The project received a research grant from Ortomedic AS and Eckbos legate.

Handling co-editors: Keijo Mäkelä and Robin Christensen

*Acta* thanks Thomas Jakobsen and Klaus Kjær Petersen for help with peer review of this manuscript.
